# Vemurafenib Induces Senescent Phenotype with Increased Adhesion in BRAF Mutant A375 but not in Wild Type BRAF SK-MEL-2 Melanoma Cells

**DOI:** 10.34172/apb.42808

**Published:** 2025-02-12

**Authors:** Aleksandra Rashidovna Esimbekova, Vasiliy Dmitrievich Belenyuk, Andrey Anatolievich Savchenko, Tatiana Gennadievna Ruksha

**Affiliations:** ^1^Department of Pathophysiology, Krasnoyarsk State Medical University, Krasnoyarsk, 660022, Russia; ^2^Laboratory of Cell Molecular Physiology and Pathology, Federal Research Center, Krasnoyarsk Science Center of the Siberian Branch of the Russian Academy of Sciences, Krasnoyarsk, 660022, Russia

**Keywords:** Melanoma, Vemurafenib, Focal adhesion, Senescence, Cell cycle, G0 phase

## Abstract

**Purpose:**

The present study aimed to determine the selective effects of BRAF V600E inhibitor on focal adhesion in melanoma cells with respect to their phenotypic reprogramming.

**Methods:**

Flow cytometry was used to analyse the distribution of BRAFV600E and BRAFWT melanoma cells throughout the cell cycle post-vemurafenib treatment. Senescent cells were identified based on b-galactosidase activity and the mRNA expression of cell cycle proteins, CCND1 and RBL1. Centrifugal cell adhesion assay was used to determine the adhesive capacities of resting and proliferative BRAF mutant and BRAF wild-type melanoma cells under vemurafenib treatment. Fibronectin binding was evaluated by spectrophotometry and quantitative real-time PCR to measure the mRNA levels of integrins: ITGAV, ITGA5, ITGB1 and ITGB3.

**Results:**

Vemurafenib increases the proportion of melanoma BRAFV600E-positive cells in the G0 phase of a cell cycle. Melanoma cells entering the G0 phase after vemurafenib treatment indicated an upregulation of senescence-associated markers. Non-proliferating melanoma cell number was elevated among vemurafenib-treated BRAFV600E cells with enhanced attachment. BRAFV600E-positive but not BRAFV600E-negative cells were characterised by upregulated ITGAV.

**Conclusion:**

The current results demonstrated that vemurafenib induces the phenotypic switch in melanoma cells depending on their mutational status. It also strengthens the adhesive features of senescent cells, increasing their binding to fibronectin via ITGAV, which may be a part of the phenotypic mode of drug resistance or slow interaction of proliferating cancer cells with the extracellular matrix (ECM). Thus, targeting senescent cells by focal adhesion modulators may be a promising approach to control drug-resistant melanoma cells.

## Introduction

 Cutaneous melanoma is a result of the neoplastic transformation of melanocytes and is the most lethal cancer among cutaneous malignancies. About 50% of cutaneous melanomas contain activated V600 mutations in the *BRAF* gene encoding serine-threonine protein kinase BRAF.^1^ In 2011, the Food and Drug Administration (FDA) approved PLX4032, also known as vemurafenib, for the treatment of melanoma.^2^ Small molecule BRAF inhibitor vemurafenib (PLX4032) has revolutionised melanoma therapy by inducing rapid tumour regression. However, > 50% of patients treated with vemurafenib monotherapy experience tumour recurrence within 6-7 months after the treatment.^3,4^

 Emerging drug resistance poses a significant problem to effective treatment in oncology. The ability of tumour cells to enter the G_0_ phase of the cell cycle, rendering them resistant to therapy, considered as one of the drug resistance mechanisms.^5^ Most antitumour drugs target rapidly proliferating cells and are ineffective against G_0_-positive cells corresponding to quiescent/senescent cells.^6-8^ The G_0_ phase may harbour both reversibly quiescent and senescent cells. Quiescent cells are characterised by diminished metabolic activity and limited antigenic repertoire, whereas senescent cells exhibit specific pro-inflammatory senescence associated secretory phenotype. Cellular senescence is characterised by the specific activity of senescence-associated β-galactosidase (SA-β-gal). Senescent cells via release of senescence associated mediators remodel the tumour microenvironment, promoting a pro-inflammatory microenvironment.^9^

 A previous study showed that targeted therapy alters the secretome of tumour cells exposed to drug stress, thereby developing resistance.^10^ Such resistant cancer cells can reside in distant organs and later may promote metastasis formation. This latent state is known as ‘tumour dormancy’ and can last for decades. Non-proliferating or slow-cycling cancer cells interact with their microenvironment. Indubitably, microenvironment is a key modulator of tumour progression; thus, it is essential to assess the role of extracellular matrix (ECM) components in the development of drug resistance and tumour progression. The ECM represents the boundary between the tumour metastatic niche and normal tissue and can exert both tumorigenic and antitumour effects. Fibronectin is one of the key components of the ECM implicated in metastatic niche formation.^11^ Accumulating evidence shows that cancer cell adhesion to ECM proteins, including fibronectin, contributes to drug resistance. Cell adhesion-mediated drug resistance has been described in various tumours including multiple myeloma,^12,13^ lung cancer^14^ and uveal melanoma.^15^ As tumour progression is influenced by microenvironment, communication between cancer cells and microenvironment provided by adhesion molecules influences cell proliferation, migration, invasion, drug resistance.

 In this study, we evaluated how vemurafenib affects melanoma cells percentage in different phases of a cell cycle and its ability to induce senescence in melanoma cells.

## Materials and Methods

###  Cell lines and culture conditions

 Human cutaneous melanoma cell lines BRAF wild-type SK-MEL-2 (ATCC^®^ HTB-68^TM^, Manassas, USA) and BRAF V660E-mutated A375 (ATCC^®^ CRL-1619^TM^, Manassas, USA) were cultured in DMEM (PanEko, Moscow, Russia) with 10% foetal bovine serum (FBS) (HyClone; Cytiva, Logan, USA) and 1% antibiotic/antimycotic (Gibco; Thermo Fisher Scientific, Inc., Carlsbad, USA), in an incubator at 37 °C under 5% CO_2_ (Sanyo MSO-5AC, Tokyo, Japan). The cells were passaged at 80% confluency.

###  BRAF^V600E^ mutation analysis

 The mutational status of A375 and SK-MEL-2 melanoma cells was verified using the quantitative real-time PCR (qRT-PCR) BRAF-V600E kit (Biolink, Novosibirsk, Russia). DNA was extracted from cell cultures using the DNA extraction diaGene kit (Diam, Moscow, Russia) and estimated on a Qubit 2.0 fluorimeter (Invitrogen by Life Technologies, Singapore, Singapore) using an ssDNA HS Qubit analysis kit (Invitrogen, Eugene, OR, USA). The BRAF^V600E^ mutation was evaluated by allele-specific real time PCR, as described previously.^16^

###  Cell viability assay 

 The half-maximal inhibition (IC_50_) and maximum inhibition (2IC_50_) concentrations of vemurafenib (Selleck Chemicals LLC, Houston, USA) were evaluated in melanoma cell lines A375 and SK-MEL-2 using the MTT (3-[4,5-dimethylthiazol-2-yl]-2,5 diphenyl tetrazolium bromide) method. The MTT is converted into water-insoluble formazan by mitochondrial dehydrogenases of viable cells. Cells were plated in 96-well microtiter plates at a density of 2 × 10^4^ cells/mL and cultured overnight for cell attachment. Then, the medium was replaced with that containing 0–10 μM vemurafenib, and the culturing was continued at 37 °C under 5% CO_2_ for 72 hours. Subsequently, 5 mg/mL MTT reagent (Invitrogen, the Netherlands) was added to each well for 4 h. Finally, the reaction was quenched with 100 μL dimethyl sulfoxide (DMSO; Helicon, Moscow, Russia). The optical density was measured at 560 nm on an Efos-9305 spectrophotometer (Shvabe Photosystems, Moscow, Russia). The IC_50_ value was corresponded to 50 percent drop of metabolic activity. The assay was carried out in three biological replicates.

###  Vemurafenib treatment 

 Vemurafenib (PLX4032) was obtained from Selleck Chemicals LLC. Melanoma cells were treated with 0.45 µM and 0.9 µM for the A375 cell line, 1.7 µM and 3.4 µM for the SK-MEL-2 line corresponding to IC_50_ and 2IC_50_ of vemurafenib, respectively, for 3 days, washed with phosphate-buffered saline (PBS; Helicon, Moscow, Russia) and cultured for 48 hours. On day 5 after the treatment, the cells were harvested for further experiments. DMSO served as the negative control, the cells were treated with DMSO in an independent well concurrent to vemurafenib.

###  Cell cycle analysis 

 The distribution of cells across cell cycle phases was assessed by propidium iodide (PI) staining and monoclonal antibodies against Ki-67. Cells were treated with vemurafenib at IC_50_ and 2IC_50_ concentrations, washed with PBS (Helicon), fixed with 70% ice-cold ethanol, permeabilized with 0.1% Triton X100 (Biotechnik GmbH, Gaiberg, Germany) and treated with RNase A (100 μg/mL) (Invitrogen, Thermo Fisher Scientific, Vilnius, Lithuania). Then, the cells were stained with anti-Ki-67 monoclonal antibodies labelled by FITC (clone: SolA15, eBioscience, Thermo Fisher Scientific, Carlsbad, USA) at a concentration of 1:100 and 100 μg/mL PI (Invitrogen, Thermo Fisher Scientific, Carlsbad, USA), followed by incubation at 37 °C in the dark for 1 h. The percentage of cells in each phase of the cell cycle was determined on a Cytomics FC-500 flow cytometer (Beckman Coulter, Fullerton, USA) and CXP software (version 2.2; Beckman Coulter, Brea, USA) based on the differences in Ki-67 expression level and RNA content of the cells, which was characterised by PI staining. Typically, cells in the G_0_ phase were characterised by diminished Ki-67 levels and RNA content, which distinguishes them from proliferating cells. Gating was carried out in the fluorescence range Ki-67-FITC ≤ 100 (negative) and in the fluorescence range PI from 0.7 to 1.3 relative units. The experiment was done in three biological replicates.

###  Immunocytochemistry 

 The immunocytochemical study was performed using the cell proliferation marker Ki-67 to determine the portion of cells residing in the G_0_ phase of the cell cycle. Cells were incubated with vemurafenib at IC_50_ and 2IC_50_ concentrations, fixed with 10% formaldehyde and permeabilized with 0.5% Triton X-100 (Biotechnik GmbH, Gaiberg, Germany). Then primary rabbit monoclonal antibodies to human Ki-67 (1:100; ab15580; Abcam, Cambridge, USA) in 10% FBS (HyClone, GmbH, Parsching, Austria) were applied at 4 °C overnight. Secondary goat anti-rabbit antibody labelled with Alexa Fluor 488 (Invitrogen, Thermo Fisher Scientific, Eugene) were used at a dilution 1:200 for 1 h. DAPI (AppliChem GmbH, Darmstadt, Germany) at concentration 1 μg/mL was used for 15 min. The proportion of Ki-67-positive cells was evaluated in at least ten fields on a FLoid Cell Imaging Station (Thermo Fisher Scientific, Bothell, USA). Proliferating cells were considered as cells with nuclei stained green and blue, whereas non-proliferating G_0_-positive cells were characterised by blue nuclei.

###  Detection of β-galactosidase activity

 In order to determine the level of vemurafenib-induced senescent melanoma cells, the activation of β-galactosidase was evaluated. This cytochemical analysis was based on staining the cells containing the active enzyme with the chromogen X-gal (5-bromo-4-chloro-3-indoyl β-d-galactopyranoside). The cells were cultured with vemurafenib at IC_50_ and 2IC_50_ concentrations, fixed with 4% formaldehyde and stained in the dark at 37 °C. The staining buffer included 1X citric acid/sodium phosphate buffer (pH 6.0) (Abcam, UK), 5 mmol potassium hexacyanoferrate (III) (AO Reakhim, Moscow, Russia), 5 mmol potassium hexacyanoferrate (II) (AO Reakhim), 150 mmol NaCl (JSC Reakhim), 2 mmol MgCl2 (JSC Reakhim) and 1 mg/mL 5-bromo-4-chloro-3-indolyl β-D-galactopyranoside (Invitrogen; Thermo Fisher Scientific). X-gal undergoes hydrolysis in the presence of active β-galactosidase to form a blue product visualised under a microscope (MIB-R; LOMO-Microsystems, Saint Petersburg, Russia).

###  Centrifugal cell adhesion assay

 Vemurafenib-treated melanoma cells were cultured at a density of 2 × 10^5^ cells/mL in the culture flasks. Then they were filled with PBS and subjected to centrifugation upward in a monolayer at 1000 rpm for 3 min. Then the cells were washed with PBS, fixed with 10% formaldehyde and permeabilized with 0.5% Triton X-100 (Biotechnik GmbH, Gaiberg, Germany). Then cells were subjected to incubation with anti-Ki-67 monoclonal antibodies (1:100; ab15580; Abcam) overnight at 4 °C followed by the application of a secondary IgG goat antibody conjugated with Alexa Fluor 488 (H + L) (1:100; Invitrogen, Thermo Fisher Scientific, Eugene, USA) at room temperature in the dark for 90 min. Cells were incubated with DAPI (1:10 000) for 15 minutes. The percentage of Ki-67-negative cells was assessed on a Floyd Cell Imaging Station. Ki-67-negative cells were characterised by blue nuclei.

###  Adhesion to fibronectin

 A fibronectin human plasma solution (100 μg/mL) (Sigma-Aldrich, St. Louis, USA) was dispensed into a 96-well plate to create an adhesive substrate, while wells without coating served as controls. The wells were rinsed with sterile PBS before seeding the cells at a density of 3 × 10^4^ cells/mL for 2.5 hours in a CO_2_ incubator; the nonadherent cells were discarded, and the cells were washed with PBS before staining with methylene blue. The optical density of adherent fibronectin cells was measured at 620 nm on an Efos-9305 spectrophotometer (Shvabe Photosystems, Moscow, Russia).

###  qPCR real time

 Total RNA was isolated from cells using the RNA isolation kit (Diam, Moscow, Russia). The RNA concentration was measured on a Qubit 2.0 fluorimeter (Invitrogen by Life Technologies, Singapore) using the HS Qubit RNA analysis kit (Invitrogen, Eugene, Oregon, USA). Moloney murine leukaemia virus (MMLV) RT kit (Eurogen, Moscow, Russia) was used for cDNA synthesis by reverse transcription reaction. The qRT-PCR reaction mixture consisted of a 2.5X Master Mix with ROX (Syntol, Moscow, Russia), deionised water and 20X primer TaqMan^TM^ Gene Expression Assay: CCND1 Hs00765553_m1, RBL1 Hs00765700_m1 (cat. no. 4331182, Applied Biosystems, Pleasanton, USA). The primers for adhesion molecules expression evaluation were as follows: *ITGA5* (Ensembl: ENSG00000161638), *ITGAV* (Ensembl: ENSG00000138448), *ITGB1* (Ensembl: ENSG00000150093) and *ITGB3* (Ensembl: ENSG00000259207). The amplification was carried out on a Step One^TM^ real-time PCR device (Applied Biosystems, Singapore, Singapore). Amplification regime included 50 °C for 2 min, 95 °C for 10 min and 40 cycles at 95 °C for 15 s and 0 °C for 1 min. *GAPDH* (Ensembl: ENSG00000111640) and *HPRT* (Ensembl: ENSG00000165704) were used as endogenous controls. All the aforementioned primers were synthesised by DNA Synthesis Ltd (Moscow, Russia). The data were analysed with the use ΔΔCt method. The assay was carried out in three technical replicates.

###  Statistical analysis

 Statistical analyses were conducted using Statistica 7.0 (StatSoft, Inc., Statistics, USA). GraphPad Prism (v8; GraphPad Software, Inc.; https://www.graphpad.com/) was used to generate the graphs. *P* < 0.05 was considered as statistically significant. All the experimental procedures were conducted in three biological replicates.

## Results

**Figure 1:**
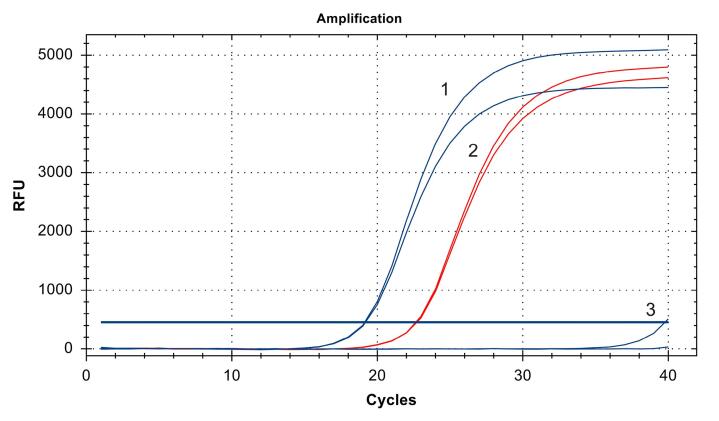
Mutational status of A375 and SK-MEL-2 melanoma cells determined by allele-specific real-time PCR. The Ct value of DNA isolated from A375 cells was 19.15 (1), which was similar to the Ct value of the positive control (2), whereas the Ct value of DNA isolated from SK-MEL-2 cells was 39.77 (3)

 Allele-specific real-time PCR determined that the Ct value for DNA isolated from SK-MEL-2 cells was 39.77, whereas that of the DNA isolated from A375 cells was 19.15, similar to the positive control DNA. Therefore, we speculated that A375 melanoma cells harbour *BRAF* V600E mutation, whereas SK-MEL-2 cells characterised by wild-type *BRAF* (Figure 1).

**Figure 2:**
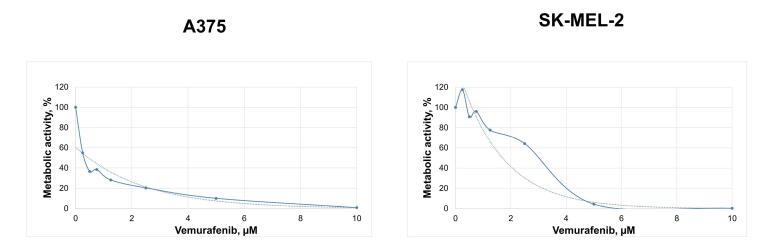
Changes in the metabolic activity of cutaneous melanoma cells BRAF^mt^ A375 and BRAF^wt^ SK-MEL-2 depending on the concentration of vemurafenib demonstrated by the MTT assay

 The MTT assay showed that BRAF^mt^ A375 and BRAF^wt^ SK-MEL-2 vemurafenib treatment decreased the metabolic activity with increasing vemurafenib concentration. IC_50_ and 2IC_50_ of vemurafenib were 0.45 µM and 0.9 µM for the A375 melanoma cells and 1.7 µM and 3.4 µM for the SK-MEL-2 cells, respectively (Figure 2).

 Then, vemurafenib at concentrations IC_50_ and 2IC_50_, was added to A375 and SK-MEL-2 to determine the effects of the drug on cell cycle phase distribution by flow cytometry using PI and anti-Ki-67-FITC monoclonal antibodies.

**Figure 3:**
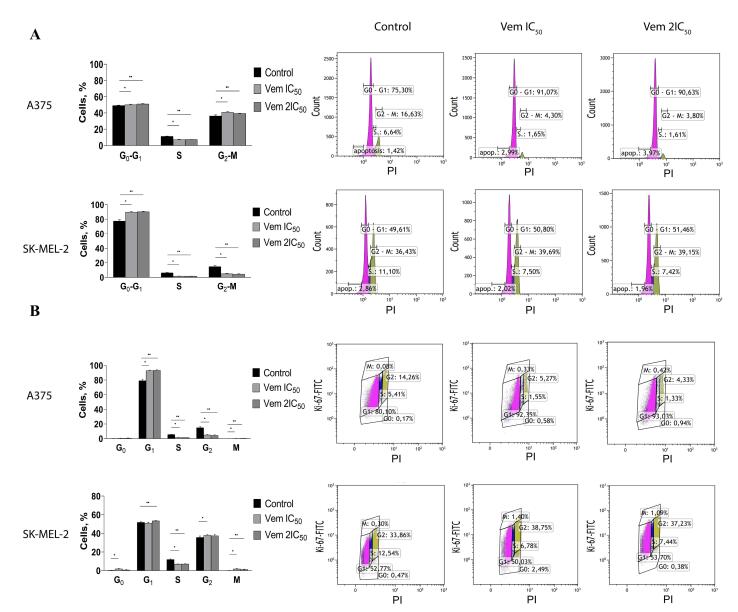
Changes in cell ratio corresponding to the cell cycle phase under the influence of vemurafenib IC_50_ and 2IC_50_ concentrations on BRAF^mt^ A375 and BRAF^wt^ SK-MEL-2 cutaneous melanoma cells shown by the results of flow cytometry with PI (A). Changes in the ratio of cells corresponding to the phase of the cell cycle under the vemurafenib IC_50_ and 2IC_50_ concentrations on cutaneous melanoma cells of the BRAF^mt^ A375 and BRAF^wt^ SK-MEL-2 cell lines according to the results of flow cytometry with Ki-67-FITC and PI (B)

 The level of apoptotic *BRAF*^mt^ A375 cells corresponding to cells residing in pre-G_0_ phase increased by 2.3 times after treatment with 0.45 μM vemurafenib (IC_50_) and by 2.4 times after 0.9 µM vemurafenib (2IC_50_). Conversely, 1IC_50_ and 2IC_50_ concentrations of vemurafenib induced a 1.7- and 1.5-fold decrease, respectively, in apoptotic cells in *BRAF*^wt^ SK-MEL-2 cell line. The flow cytometry analysis of PI-stained cells showed an increased proportion of BRAF^mt^ A375 cells residing in G_0_-G_1_ phases from 77.42% to 89.81% and 90.28% in response to vemurafenib treatment at 0.45 µM and 0.9 µM, respectively, whereas the cell percentage was decreased in the G_2_-M and S phases. On the other hand, vemurafenib decreased the percentage of cells in the S phase and slightly increased the proportion of *BRAF*^wt^ SK-MEL-2 cells in the G_2_-M phase (Figure 3A).

 Ki-67 staining with subsequent flow cytometry revealed that vemurafenib increased the proportion of BRAF^mt^ A375 cells from 79.15% to 92.92% (IC_50_) and 93.27% (2IC_50_) in the G_1_ phase, followed by 2.9 times by 1IC_50_ and 3.4 times by 2IC_50_ decrease in the G_2_ phase of the cell cycle (Figure 3B).

**Figure 4:**
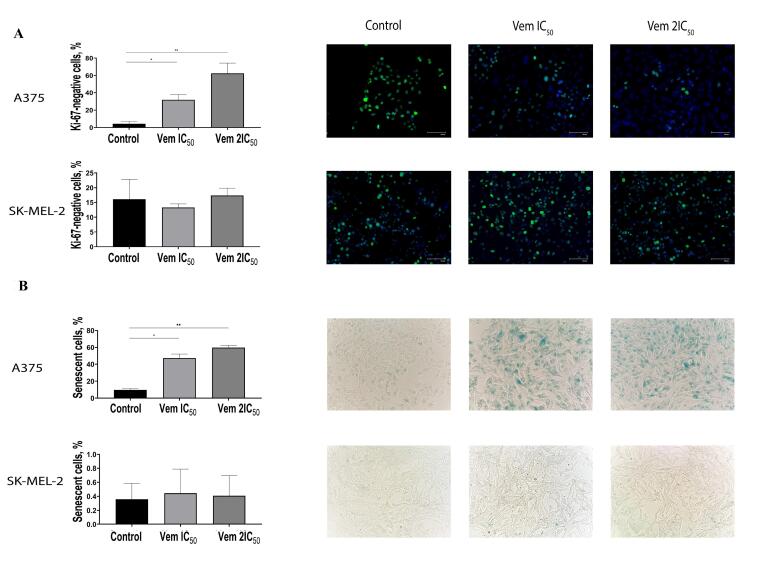
Immunocytochemistry of cutaneous melanoma cell lines BRAF^mt^ A375 and BRAF^wt^. SK-MEL-2 stained with Ki-67 antibodies and DAPI after exposure to vemurafenib concentrations IC_50_ and 2IC_50_, wherein the nuclei of Ki-67-positive cells are green, and the nuclei of Ki-67-negative, G_0_-positive cells are blue (A). Altered proportion of BRAF^mt^ A375 and BRAF^wt^ SK-MEL-2 cutaneous melanoma cells expressing β-galactosidase following exposure to vemurafenib IC_50_ and 2IC_50_ concentrations, wherein β-galactosidase-positive cells, termed senescent cells, are visualised as blue coloured (B). Note: * *P* < 0.05 – statistically significant differences between cells exposed to vemurafenib IC_50_ concentration and control. ** *P* < 0.05 – statistically significant differences between cells exposed to vemurafenib concentration 2IC_50_ and control.

 The visualisation of Ki-67 staining revealed a significant increase in the percentage of Ki-67-negative BRAF^mt^ A375 cells that corresponded to G_0_-positive cells: from 4.31% to 31.82% after treatment with 0.45 μM vemurafenib (IC_50_) and 62.46% after treatment with 0.9 μM vemurafenib (2IC_5_0). However, flow cytometry showed an elevation in the cells corresponded to the G_1_ phase. Interestingly, the variable expression of Ki-67 in the G_1_ makes it challenging to differentiate the G_1_ and G_0_ phases.^17^ Immunocytochemistry showed an increase in the percentage of G_0_-positive BRAF^mt^ A375 cells, while that of G_0_-positive BRAF^wt^ SK-MEL-2 cells remained unchanged (Figure 4A).

**Figure 5:**
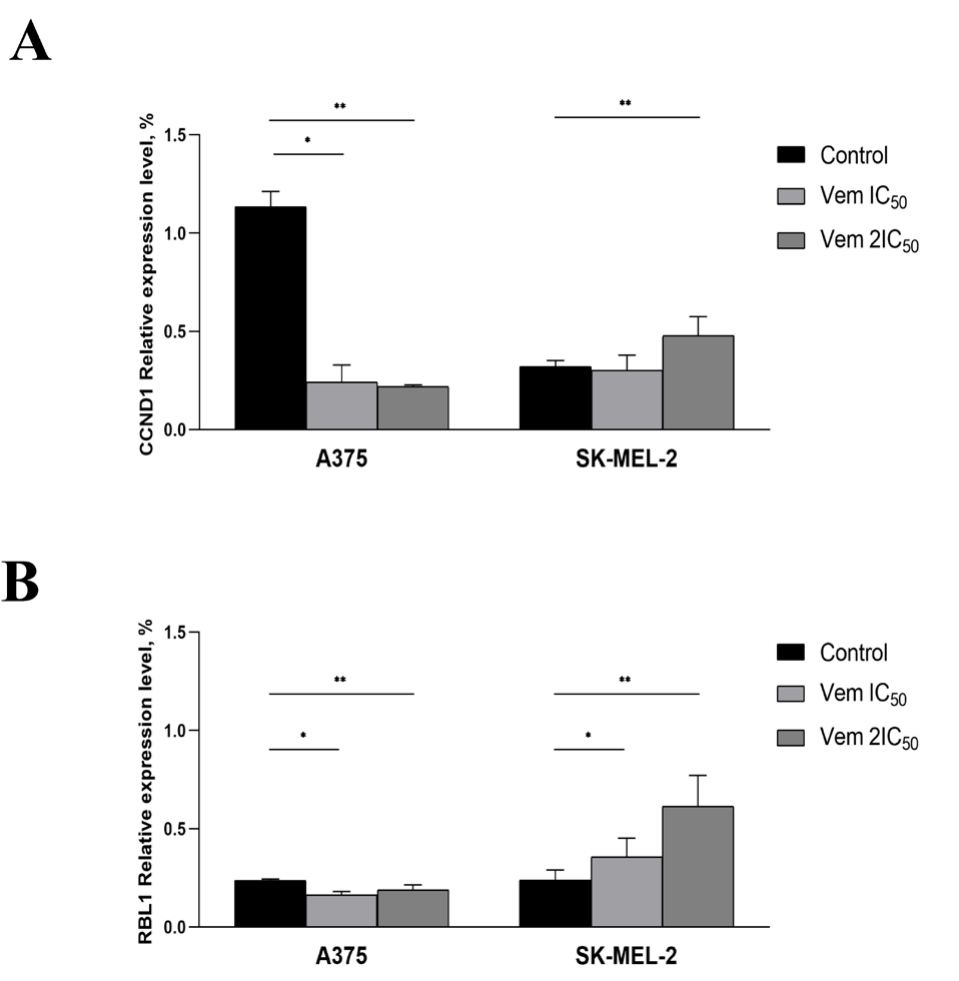
Relative *CCND1 *(A) and *RBL1 *(B) gene expression levels under vemurafenib treatment at 0.45 µM (IC_50_) and 0.9 µM (2IC_50_) for A375 cell line, 1.7 µM (IC_50_) and 3.4 µM (2IC_50_) for the SK-MEL-2 melanoma cells. Note: * *P* < 0.05 – statistically significant differences between cells exposed to vemurafenib at IC_50_ concentration and control. ** *P* < 0.05 – statistically significant differences between cells exposed to vemurafenib 2IC_50_ concentration and control

 The G_0_ phase of the cell cycle consists of both quiescent cells that retain the ability of proliferation and senescent cells that characterized by permanent exit from a cell cycle followed by elimination through apoptosis. Then, the cells were subjected to 5-bromo-4-chloro-3-indolyl β-D-galactopyranoside, a substrate for β-galactosidase. The positive cells were stained blue (Figure 4B). Under 0.45 μM (IC_50_) vemurafenib treatment, the rate of senescent (β-galactosidase-positive) BRAF^mt^ A375 cells was increased from 9.75% to 47.24%, whereas 0.9 μM (2IC_50_) vemurafenib increased the cell content up to 59.86%; however, the level of β-galactosidase-positive BRAF^wt^ SK-MEL-2 cells under vemurafenib treatment remained unchanged. In order to confirm the transition of the melanoma cells to a senescent state under vemurafenib treatment, we analysed the expression and evaluated the mRNA levels of cyclin D1 (*CCND1*) and *RBL1*. The results showed decreased levels of *CCND1* and *RBL1* in A375 cells. Instead, SK-MEL-2 cells were characterised by upregulated levels of both molecules (Figure 5).

**Figure 6:**
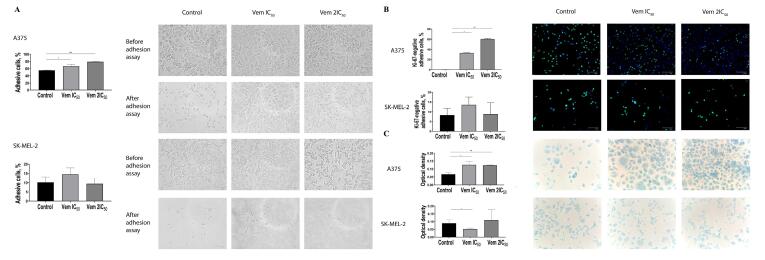
Adhesive capacities of cutaneous melanoma BRAF^mt^ A375 and BRAF^wt^ SK-MEL-2 cells after exposure to vemurafenib at concentrations corresponding to IC_50_ and 2IC_50_ determined by centrifugal force application (A). Immunocytochemical study of adherent cutaneous melanoma cells line BRAF^mt^ A375 and BRAF^wt^ SK-MEL-2 after treatment with vemurafenib corresponding to the IC_50_ and 2IC_50_ concentrations after centrifugal force application (B). Adhesion of BRAF^mt^ A375 and BRAF^wt^ SK-MEL-2 cutaneous melanoma cells to fibronectin after treatment with vemurafenib corresponding to IC_50_ and 2IC_50 _concentrations (C). Note: * *P* < 0.05 – statistically significant differences between cells exposed to dacarbazine at IC_50_ concentration and control. ** *P*< 0.05 – statistically significant differences between cells exposed to vemurafenib concentration 2IC_50_ and control

 Furthermore, an increase in the percentage of G_0_-positive senescent BRAF^mt^ A375 cells was correlated with increased cell adhesion abilities, as determined by enhanced adhesion to the surface of the culture flask under centrifugation (Figure 6A). 0.45 μM vemurafenib (IC_50_) and 0.9 μM vemurafenib (2) treatment of BRAF^mt^ A375 cells increased the percentage of adherent cells from 55.27% to 66.6% and 79.05% after centrifugation. Also, an increased proportion of G_0_-positive cells was observed in melanoma cells attached to the bottom of the flask post-centrifugation. The percentage of attached Ki-67-negative cells was 0.13% cells in the control and was 32.74% in 0.45 μM vemurafenib-treated cells and 60.19% in 0.9 μM vemurafenib-treated cells (Figure 6B). BRAF-negative SK-MEL-2 cells exhibited neither an increase in senescent cell proportion under vemurafenib treatment nor an increase in Ki-67-negative cells. The value of the optical density of BRAF-positive A375 melanoma cells attached to fibronectin was increased 1.87 times, whereas BRAF-negative SK-MEL-2 cells did not show any alteration in adhesion to fibronectin after vemurafenib treatment (Figure 6C).

**Figure 7:**
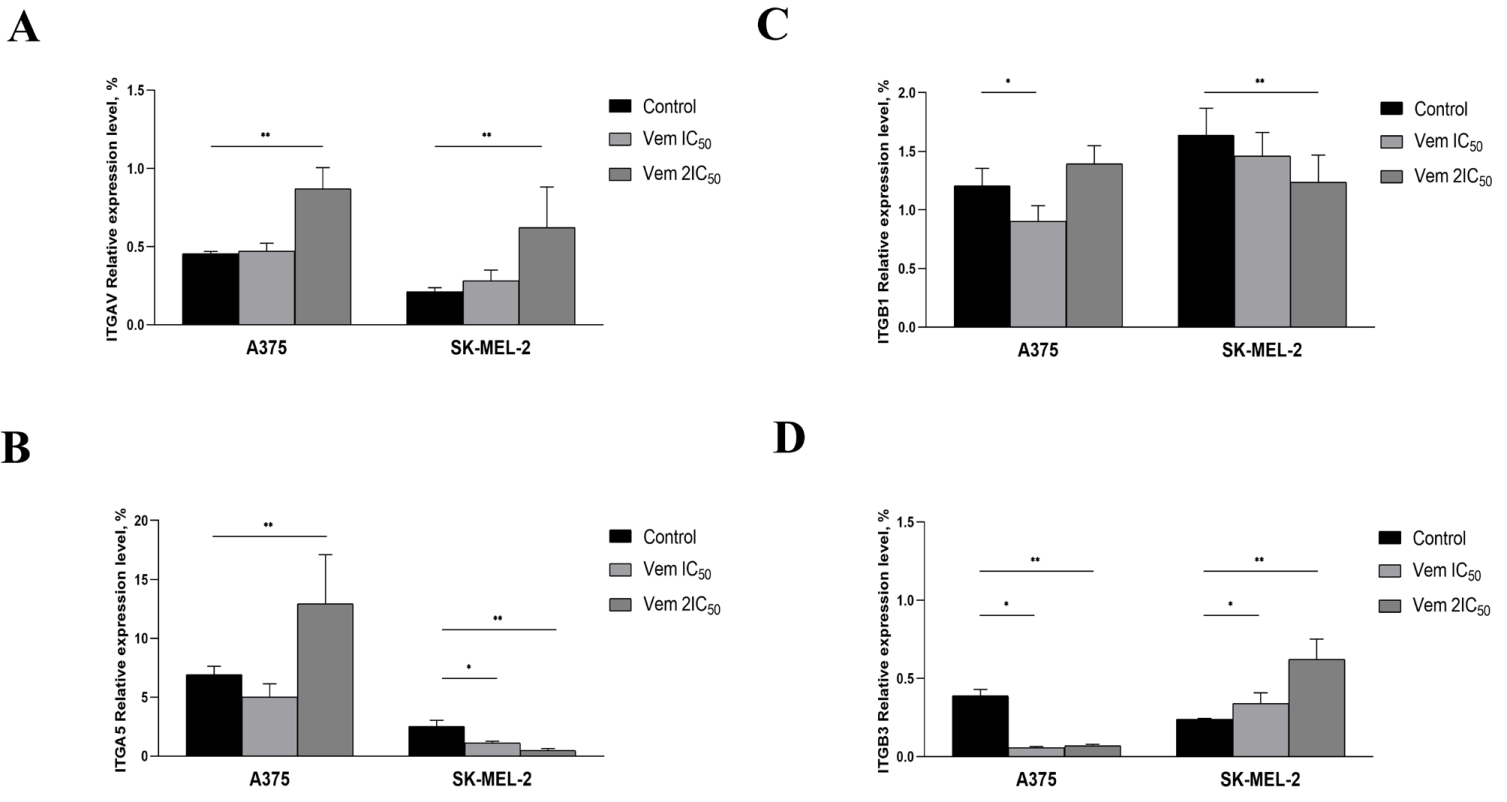
Altered expression levels of*ITGAV *(A), *ITGA5* (B), *ITGB1 *(C) and *ITGB3 *(D) under vemurafenib treatment at 0.45 µM (IC_50_) and 0.9 µM (2IC_50_) for A375, 1.7 µM (IC_50_) and 3.4 µM (2IC_50_) for SK-MEL-2. Note: * *P* < 0.05 – statistically significant differences between cells exposed to vemurafenib concentration corresponding to IC_50_ concentration and control. ** *P* < 0.05 – statistically significant differences between cells exposed to vemurafenib concentration corresponding to 2IC_50_ and control

 Cancer cells interact with fibronectin via integrins: ITGAV, ITGA5, ITGB1 and ITGB3. Therefore, the levels of these integrins were evaluated in A375 and SK-MEL-2 after vemurafenib exposure. Consequently, 2IC_50_ vemurafenib upregulated ITGAV and ITGB3 mRNA levels in both A375 and SK-MEL-2 cells but downregulated ITGB1 and ITGB3 expression levels in A375. ITGA5 was increased in A375 but decreased in SK-MEL-2 cells post vemurafenib treatment at IC_50_ and 2IC_50_ concentrations (Figure 7).

 Thus, BRAF-positive melanoma cells that remained viable after exposure to vemurafenib exhibited characteristics of senescent tumour cells and increased adhesive properties due to fibronectin binding. Wild-type *BRAF* gene harbouring melanoma cells exhibited neither the characteristics of senescent cells nor changes in adhesion levels.

## Discussion

 Melanoma resistance to molecular targeted therapy was observed after implementing BRAF inhibitors into clinical practice. However, several mechanisms have been associated with melanoma drug resistance to vemurafenib. Thus, vemurafenib-resistant cells characterised by mitogen-activated protein kinase (MAPK) reactivation through expression of aberrantly spliced mutated BRAF protein exhibiting kinase activity.^18^ Also, other mechanisms of melanoma BRAF resistance underlie BRAF amplification.^19^ and the activation of the prooncogenic signalling pathway.^20^

 Melanoma is recognised as a highly plastic tumour system, wherein multiple processes are activated in response to anticancer drug application. Cancer cell plasticity is a mechanism for phenotype modification to adapt and survive in an unfavourable environment for further progression and metastasis. A recent study showed that cancer cells escape immune evasion and apoptosis-inducing stimuli by transient transition from the cell cycle to the quiescent state corresponding to the G_0_ phase of the cell cycle.^21^ Thus, understanding cell cycle associated resistance mechanisms is essential to developing effective personalised, targeted therapies.

 Vemurafenib blocks the activation of MAPKs with V600E mutation through ATP-competitive inhibition of the kinase domain of BRAF. Although vemurafenib is more specific than cytotoxic chemotherapeutic agents, it may also exert off-target effects.^22^ In the current study, vemurafenib inhibits the proliferative activity of BRAF^WT^ SK-MEL-2 cells but at a concentration four times higher than in BRAF^V600E^ A375 cells.

 Our results showed that vemurafenib induces exit from the cell cycle concurrent to shifting BRAF-positive melanoma cells from proliferative to senescent state that was evaluated by histochemical assay detecting the β-galactosidase activity and the altered expression levels of cell cycle-related proteins — CCND1 and RBL1. CCND1 interacts with cyclin-dependent kinase 4 or 6, followed by retinoblastoma protein inactivation essential for cell cycle transition from the G_1_ to the S phase.^23^ As observed previously, we also showed that CCND1 depletion is associated with cancer cell transition to a senescent state.^24^ The hyperphosphorylated form of RBL1 interacts with the transcription factors to repress the transcription of genes implicated in cell cycle progression.^25^ Moreover, RBL1 downregulation is linked to the transition to the senescent state.^26^ Therefore, both cell cycle-related genes — *CCND1* and *RBL1 *— exhibited altered expression patterns accompanying a shift to the senescent state.

 Senescence is defined as permanent cell cycle arrest followed by apoptosis development. However, recent studies found that the senescent state is a reversible condition. It is in the line with our study indicated that melanoma cells resistant to the proapoptotic effect of vemurafenib are characterised by reduced proliferation and increased β-galactosidase activity, which are characteristics of senescent cells. Senescence facilitates stress-responsive cancer cells to adapt to altering environmental conditions primarily via epigenetic reprogramming.^27^ Our previous studies revealed that dacarbazine-induced melanoma cells exit from the cell cycle accompanied by transcriptional activation of focal adhesion signalling and enhanced adhesive capacities.^24^ Additionally, resting BRAF-positive melanoma cells treated with BRAF inhibitor exhibited similar phenotypic features. In the present study, we observed that G_0_-positive but senescent melanoma cells adhere to surfaces post-centrifugation. Moreover, these cells exhibit facilitated interaction with fibronectin. Strikingly, the adhesive capacities and interaction with fibronectin remained unchanged in vemurafenib-treated BRAF-negative melanoma cells.

 ECM is a critical regulator of disseminated cancer cells’ reprogramming during their migration, intra- and extravasation and further seeding in distant organs. However, whether ECM supports quiescent cell reactivation is yet to be clarified. Fluegen et al showed that hypoxic ECM favours the shift of proliferative cancer cells to a quiescent state.^28^ Urokinase receptor interaction with alpha-5 beta-1 integrin stimulates cancer cell adhesion to fibronectin, stimulating ERK-mediated cell proliferation.^29^ Thus, increased adhesive features in senescent melanoma cells represent phenotypic reprogramming and essential features to maintain the quiescent state or balance between proliferative and cell arrest stimuli.

 Among ECM components, fibronectin is a key player that establishes the premetastatic niche.^11^ It is involved in tumour progression, drug resistance and metastasis development in various solid tumours.^30-32^ Fibronectin interaction with circulated cancer cells via focal adhesion protein Talin1 facilitates the formation of a premetastatic niche in the liver^33^ and promotes epithelial-mesenchymal transition, migration and invasion in MCF-7 breast cancer cells.^34^ A recent study established that fibronectin-dependent compression of tumour cells by cancer-associated fibroblasts reduces the transcription factor YAP-mediated cancer cell proliferation.^35^ ITGAV (also known as CD51) is shown to be a marker of colorectal cancer stem cells. In addition, ITGAV-positive cancer cells demonstrated chemoresistance to 5-fluorouracil (5-FU) and oxaliplatin.^36^ This observation was in line with the current results showing increased ITGAV levels in viable BRAF-positive melanoma cells after vemurafenib treatment. On the other hand, ITGAV triggers the transforming growth factor-beta following epithelial-mesenchymal transition activation. This phenomenon corresponded to our previous results that non-apoptotic BRAF-positive primary melanoma cells were characterized by enhanced migratory and invasive capacities after vemurafenib treatment.^16^ Recent data presumed that senescent cells have the potency to return to the proliferative state.^37^ Moreover, non-dividing cancer cells express various phenotypes, resulting in a heterogeneous population of slow-cycling cells^16^

 Taken together, the current observations provide an in-depth insight into vemurafenib-associated cancer cell resistance, thereby enhancing our understanding of tumour cell plasticity induced by anticancer agents.

## Conclusion

 Vemurafenib induces senescence in BRAF^V600E^ melanoma cells, avoiding apoptosis. Senescent cells were characterised by increased adhesive capacities and binding to fibronectin that corresponds to ITGAV expression increase. Our findings reflect the phenotypic drug-resistance and non-proliferating cancer cell interaction with the ECM matrix for survival. Thus, targeting senescent cells by focal adhesion modulators can be considered further as possible approach to optimize treatment modalities in melanoma patients.

## Competing Interests

 The authors declare no conflict of interest.
